# Letter from Dr. C. H. Hughes, on Electricity in Medicine

**Published:** 1887-12

**Authors:** C. H. Hughes


### Correspondence.

### LETTER FROM DR. C. H. HUGHES, OF ST. LOUIS.

EDITOR OF "THE ALIENIST AND NEUROLOGIST," ON THE SUBJECT OF ELECTRICITY IN MEDICINE.

*F. E. Daniel, M. D., Austin, Texas.*

My Dear Doctor:—In regard to Dr. F. T. Paine's discovery, he has struck a truth partially, but not entirely, a truth, which has been verified in my own clinical and therapeutical experience, for many years. *The impression of a constant galvanic current of suitably adapted intensity, and passed from center to periphery over a painfnl nerve is anodyne* always. Such a current alters the abnormal molecular activities, which underlie and are the condition of the neural pain. Such a current is not irritant, but tranquilizing, and in order not to excite pain, must be applied *without vibration*, and with wet sponge, or cotton, or other cloth electrode.

Pressure pain, or pain from neuritis, must be very cautiously treated, to be relieved by galvanism. Terminal hyperæsthesia may be so intense as not to tolerate even the weight of a wet sponge, or the slightest electrical vibration, unless well cushioned, in its approach to the affected nerve with water waves, such as are supplied in the meshes of the finest surgeon's sponge.

Pressure pain without peripheral neuritis as in inflammation is relieved through the undoubted and demonstrable influence exerted by galvanism over the vaso-motor system, and consequent blood supply of the painful nerve, while in anæmic pain the electricity, interrupted or Faradic, should be used as an irritant and excitant, the same as stimulating liniments, frictions, etc.

The principles are set forth in the monographs I send you which indicate my method of procedure for the past ten years or more.

The hyperæmia of a burn may be controlled by electrical influence over the vaso-motor system just as by the same influence a blister may be emptied of its serum, but this is not because electricity is a counter peripheral irritant, but because of its influence over the vaso-motor mechanism to restrain and tranquilize the irritation it receives from a scald or burn. The therapeutic effect is the same as observed by Dr. Paine, but it has the same influence that stops a leucorrhœa as ———* showed twenty-five years before the gynecologists were willing to believe it.

If I had time from my many other engagements, I would give you a paper on the subject at some length, but I cannot take more time.

The subject is intensely interesting, and I am glad to see that the light is dawning in the direction of practical general observation and that we, who have so long and faithfully studied the rationale of electro-neurotherapy, are not to be always looked upon as over confident cranks with handy cure-alls to offer for every ill.

Electricity is the greatest of physiological alteratives in therapeutics, the adjuvant of all other neurotherapy, and when the day comes when it is no longer despised or rejected, will be the day of

* This word is illegible.—Compositor.

the beginning of the greatest progress in therapeutics. It will never supplant a rational internal or external medical therapeutics but it will aid both.

When effusions do not even begin to disappear under iodide of potassium, elaterium or buchu, the movement may be started with suitable electrization; when the appropriating powers of the system have failed and neither arsenic, nor iron, nor quinine, nor other tonic takes a hold upon the depressed organism we wish them to, though static and galvanic electrization may awaken dormant assimilative powers and the rescue of the patient is begun.

Pernicious anæmia and congestive malarial conditions, will illustrate in practice the fact. Yours truly,

C. H. HUGHES.

